# Crown-like structures in breast adipose tissue of breast cancer patients: associations with CD68 expression, obesity, metabolic factors and prognosis

**DOI:** 10.1038/s41523-021-00304-x

**Published:** 2021-07-22

**Authors:** Martin C. Chang, Zohreh Eslami, Marguerite Ennis, Pamela J. Goodwin

**Affiliations:** 1 University of Vermont Cancer Center, Burlington, VT USA; 2 Department of Pathology & Laboratory Medicine, Larner College of Medicine at the University of Vermont, Burlington, VT USA; 3 Department of Laboratory Medicine and Pathobiology, University of Toronto, Toronto, ON Canada; 4 Applied Statistician, Markham, ON Canada; 5 Department of Medicine, University of Toronto, Toronto, ON Canada; 6 Lunenfeld-Tanenbaum Research Institute, Sinai Health System, Toronto, ON Canada

**Keywords:** Risk factors, Breast cancer, Cancer metabolism

## Abstract

Crown-like structures of the breast (CLS-B), defined by the clustering of macrophages (identified using CD68 immunohistochemical staining) to surround a dying adipocyte, are a sign of adipose-tissue inflammation. In human cohorts, CLS-B positively correlates with older age, obesity, dyslipidemia and higher levels of glucose, insulin, C-reactive protein and IL-6. In an existing cohort of early-stage breast cancer patients, CLS-B were identified using H&E stained histologic sections (hCLS-B), and by CD68 immunohistochemistry (CD68 + CLS-B). We examined associations of H&E and CD68-detected CLS-B with clinicopathologic features using *χ*^2^ tests, with metabolic factors using Wilcoxon rank sum tests and with disease free and overall survival using Cox regression models. hCLS-B were detected in 59 of 163 patients with slides (36.2%) and CD68 + CLS-B in 37 of 119 patients with paraffin blocks (31.1%). hCLS-B were positively correlated with higher weight (*p* = 0.003), BMI (*p* = 0.0008) and C-reactive protein (*p* = 0.045). CD68 + CLS-B were positively correlated with higher weight (*p* = 0.006), BMI *p* = 0.001), leptin (*p* = 0.034), insulin (*p* = 0.008) and Homeostasis Model Assessment (*p* = 0.027). CD68 + CLS-B were associated with poor distant disease-free with a hazard ratio (HR) of 2.81, 95% confidence interval (CI) 1.20–6.57, and overall survival with HR 3.97 (1.66–9.48), while hCLS-B were not associated with either: HR for distant recurrence 0.59 (0.26–1.30); HR for death 1.04 (0.50–2.16). The presence of hCLS-B and of CD68 + CLS-B were associated with obesity; CD68 + CLS-B were associated with insulin resistance and adverse prognosis. Similar patterns were not seen for hCLS-B. Research is needed to understand the biologic basis for these differences.

## Introduction

Obesity is linked to poor prognosis in breast cancer^1–3^—an effect that can be seen in all intrinsic subtypes of breast cancer. This effect is multifactorial and includes pathways mediated by systemic changes in metabolism including insulin resistance and altered adipokines^4^, and by localised tissue inflammation^5^. In mouse models high body mass index (BMI) is associated not only with a higher volume of adipose tissue, but also with alterations in adipokines (higher leptin, lower adiponectin) and increases in pro-inflammatory cytokines (e.g. TNF-α, IL-1β, Cox-2) within the adipose tissue^6^. This adipose tissue inflammation has a prominent macrophage component^6^, and potentiates both aromatase activity^7^ and downstream oestrogen-receptor-dependent gene expression underlying breast cancer growth^8^. Macrophage polarisation has also been found in adipose tissue, as seen through distinct populations of pro-inflammatory (M1) and anti-inflammatory (M2) macrophages^9^. In human tissues, in vivo measurements of macrophage-linked inflammatory factors has not been feasible, although macrophages are readily appreciated using CD68 immunohistochemistry^10^

Much of the research to date into the role of macrophages in breast cancer has focused on the tumour microenvironment. A recent meta-analysis^11^ of the prognostic significance of tumour-associated macrophages (TAMS) reported a high density of TAMS to be associated with reduced disease free and overall survival (DFS, OS), with hazard ratios (HRs, 95% CI) of 1.5 (1.20–1.88) and 2.2 (1.72–2.89) respectively for high versus low density of TAMs. High density of TAMS was also associated with adverse tumour characteristics. These prognostic associations are most apparent in triple negative^12,13^ and HER2 positive^13^ breast cancer.

Recent research has focused on macrophages and associated inflammatory changes in breast adipose tissue that is not closely associated with tumour cells. Crown-like structures of the breast (CLS-B), defined by the clustering of macrophages (typically identified using CD68 staining, a pan-macrophage marker) to surround a dead/dying adipocyte^14,15^ are a sign of adipose-tissue inflammation. In human cohorts, the presence of CD68-positive CLS-B in both prophylactic mastectomy specimens and cancer resections has been positively correlated with older age, higher BMI, postmenopausal status, dyslipidemia, and higher levels of glucose, insulin, leptin (and lower adiponectin), C-reactive protein and IL-6^15^. In a retrospectively analysed cohort of breast cancer patients with known recurrence or metastases, CLS-B were more frequently seen in those patients with poorer survival^15^; however, prognostic data in prospective cohorts are conflicting^16,17^. The presence of CLS-B in patients with benign breast disease has also been associated with higher breast cancer risk in both female^18^ and male^19^ patients.

We have previously characterised the body size and metabolic status of a cohort of breast cancer patients enroled in a prospective study of obesity and breast cancer outcomes^20,21^. This cohort represents an opportunity to evaluate CLS-B in breast adipose tissue in a breast-cancer population with known body mass index (BMI), blood markers of metabolism and inflammation, and outcome after over 10 years of follow-up. The objective of the current study was to use available archival tissues to examine breast adipose tissue for CLS-B, with an emphasis on comparing CD68 immunohistochemistry to standard histology using the hematoxylin-and-eosin (H&E) stain. The presence of CLS-B by each method was analysed in relation to patient and tumour characteristics, metabolic factors (including insulin, glucose, adipokines and hsCRP) and patient outcomes (DFS, OS).

## Results

### Cohort characteristics and CLS-B prevalence

**Fig. 1:**
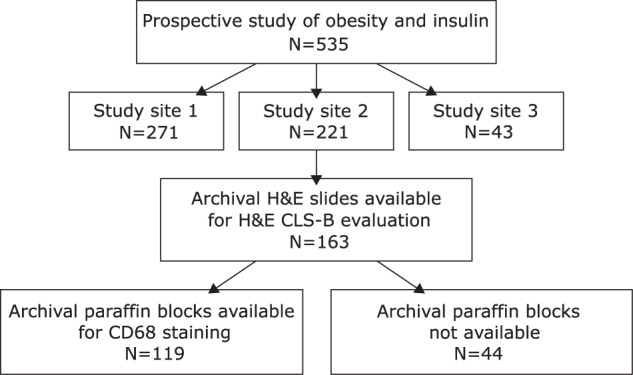
Flow diagram for patient enrolment and sample availability.

The overall cohort consisted of 163 patients from a single institution for which archival H&E slides were available (Fig. 1). Blocks for CD68 staining were available in 119 patients. Patients with and without blocks were similar with respect to age at diagnosis, BMI, surgical approach (partial vs total mastectomy), tumour grade, tumour stage, nodal stage, hormone receptor status, and presence of lymphovascular invasion and menopausal status (Supplementary Table 1).

**Fig. 2:**
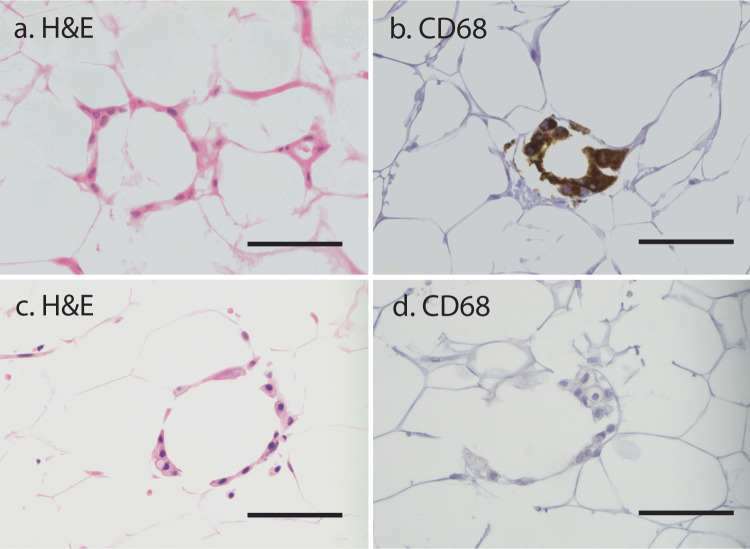
Photomicrographs of crown-like structures of the breast in representative cases. H&E CLS-B: a continuous ring of macrophages surrounding a white-fat adipocyte can be seen in H&E sections (**a**, **c**). CD68 + CLS-B: immunohistochemistry for CD68 highlights the cytoplasm of macrophages (**b**). In some cases, histologic CLS-B (hCLS-B) were CD68 negative (**d**). Colour balancing has been applied to all pixels uniformly in each individual panel. The scale bars correspond to 70 µm.

Routine H&E examination found histologic CLS-B (hCLS-B) in 59/163 cases (36%), including 46/119 (39%) in those with blocks (Table 1). In the group with blocks, CD68 + CLS-B (e.g. Fig. 2a, b) were present in 37/119 (31%) cases. These 37 cases included 22 of the cases with hCLS-B (22/46 = 47.8%) and 15 additional cases found to have CLS-B on CD68 staining but not on H&E staining (15/73 = 20.5%). Of the 46 cases with hCLS-B, 24 (52%) failed to demonstrate CD68-positivity, either because the deeper section did not contain the cells of interest or because of demonstrable CD68-negativity within the hCLS-B (Fig. 2c, d). The cells comprising hCLS-B were not distinguishable morphologically from those of CD68 + CLS-B. The association of the presence/absence of hCLS-B and CD68 + CLS-B with patient and tumour characteristics is summarised in Table 2. Patients with hCLS-B were on average slightly older than those without hCLS-B (mean age ±standard deviation (SD) 54.5 ± 10.3 vs 51.5 ± 10.4 years, *t*-test *p* = 0.08), with a somewhat more pronounced effect in patients with CD68 + CLS-B (mean age 56.8 ± 9.6 years vs 51.5 ± 9.9 years for patients with CD68 + CLS-B absent, *t*-test *p* = 0.007). Patients with CD68 + CLS-B were more likely to have undergone partial as opposed to total mastectomy (89.2% when CD68 + CLS-B present vs 72.0% when CD68 + CLS-B not present, *p* = 0.04).

**Table 1:** Presence of CLS-B detected by H&E sections (hCLS-B) versus CD68 immunohistochemistry on paraffin blocks.

		H&E slides	H&E slides		
		hCLS-B Present^a,b^	hCLS-B Absent^a,b^	Subtotal (blocks)	Totals
	CD68 + CLS-B present^b^	22	15	37	–
Paraffin block					119
	CD68 + CLS-B absent^b^	24	58	82	
No paraffin block	–	13	31	–	44
	Subtotal (H&E slides)	59	104	–	163

^a^“hCLS-B” is defined as CLS-B detected by review of hematoxylin-and-eosin (H&E) sections, without applying immunohistochemistry (see text.).

^b^Counts indicate the number of patients, broken down by whether CLS-B was detected or not, by each method.

**Table 2:** Association of hCLS-B and CD68 + CLS-B with patient clinicopathologic characteristics.

	H&E slides *n* = 163			CD68 staining *n* = 119		
	hCLS-B Present	hCLS-B Absent	*P*-value^a^	CD68 + CLS-B Present	CD68 + CLS-B Absent	*P*-value^a^
	*n* = 59 (%)	*n* = 104 (%)		*n* = 37 (%)	*n* = 82 (%)	
Menopausal status			0.27			0.08
Pre- or peri-	27 (45.8)	57 (54.8)		13 (35.1)	43 (52.4)	
Post	32 (54.2)	47 (45.2)		24 (64.9)	39 (47.6)	
Surgical treatment			0.58			0.04
Total mastectomy	13 (22)	27 (26)		4 (10.8)	23 (28.0)	
Partial mastectomy	46 (78)	77 (74)		33 (89.2)	59 (72.0)	
Tumour grade (Nottingham)			0.39			0.99
Grade 1	20 (33.9)	23 (22.1)		10 (27)	21 (25.6)	
Grade 2	19 (32.2)	44 (42.3)		14 (37.8)	30 (36.6)	
Grade 3	18 (30.5)	33 (31.7)		12 (32.4)	28 (34.1)	
Unknown	2 (3.4)	4 (3.8)		1 (2.7)	3 (3.7)	
Tumour stage			0.54			0.97
pT1	35 (59.3)	59 (56.7)		21 (56.8)	48 (58.5)	
pT2	19 (32.2)	30 (28.8)		12 (32.4)	24 (29.3)	
pT3	2 (3.4)	10 (9.6)		2 (5.4)	6 (7.3)	
Unknown	3 (5.1)	5 (4.8)		2 (5.4)	4 (4.9)	
Nodal stage			0.52			0.35
pN0	42 (71.2)	69 (66.3)		23 (62.2)	58 (70.7)	
pN1-3	17 (28.8)	35 (33.7)		14 (37.8)	24 (29.3)	
Hormone receptor			0.15			0.20
Positive	49 (83.1)	72 (69.2)		31 (83.8)	57 (69.5)	
Negative	4 (6.8)	14 (13.5)		3 (8.1)	8 (9.8)	
Unknown	6 (10.2)	18 (17.3)		3 (8.1)	17 (20.7)	
Lymphovascular invasion			0.25			0.39
Present	10 (16.9)	21 (20.2)		5 (13.5)	17 (20.7)	
Absent	34 (57.6)	46 (44.2)		22 (59.5)	38 (46.3)	
Unknown	15 (25.4)	37 (35.6)		10 (27)	27 (32.9)	

^a^*P*-value from testing the null hypothesis of no association, using *χ*^2^ tests.

### Association of hCLS-B and CD68 + CLS-B with systemic factors of metabolism and inflammation

Table 3 summarises the associations of body size and blood measurements performed in this cohort with the presence of hCLS-B and CD68 + CLS-B. The presence (vs absence) of CD68 + CLS-B was associated with higher median weight (70.0 vs 63.0 kg, *p* = 0.0055), BMI (26.8 vs 23.8 kg/m^2^, *p* = 0.001), waist circumference (86.2 vs 79.0 cm, *p* = 0.018), insulin (49.2 vs 35.0 pmol/L, *p* = 0.0084), HOMA (1.36 vs 1.00, *p* = 0.027) and leptin (18.2 vs 13.4 ng/ml, *p* = 0.034) but not with hsCRP, adiponectin, low-density lipoprotein (LDL) cholesterol, high-density lipoprotein (HDL) cholesterol or triglycerides. The presence of hCLS-B was also significantly associated with higher median weight (67.0 vs 63.0 kg, *p* = 0.0028), BMI (26.5 vs 24.0 kg/m^2^, *p* = 0.008) and waist circumference (83.0 vs 77.8 cm, *p* = 0.003) but not with circulating metabolic variables including insulin, Homeostasis Model Assessment (HOMA)^22^ and leptin. There was also an association of the presence of hCLS-B with higher median hsCRP that was of borderline significance (1.6 vs 1.0 mg/L, *p* = 0.045).

**Table 3:** Association of hCLS-B and CD68+CLS-B with body size and metabolic factors.

	H&E slides (*n* = 163)			CD68 staining (*n* = 119)		
	Median (Q1,Q3)			Median (Q1,Q3)		
	hCLS-B Present *n* = 59	hCLS-B Absent *n* = 104	*P*-value^a^	CD68 + CLS-B Present *n* = 37	CD68 + CLS-B Absent *n* = 82	*P*-value^a^
Weight (kg)	67.0 (61.8, 77.2)	63.0 (56.9, 70.0)	0.0028	70.0 (63.4, 79.1)	62.6 (57.1, 71.9)	0.0055
BMI	26.5 (23.8, 30.3)	24.0 (21.8, 27.0)	0.0008	26.8 (24.4, 30.8)	23.8 (21.9, 26.9)	0.001
Waist circumference (cm)	83.0 (77.0, 94.0)	77.8 (72.4, 86.8)	0.003	86.2 (76.5, 95)	79.0 (72.6, 87.9)	0.018
Leptin (ng/ml)	17.6 (11.9, 22.9)	13.4 (8.8, 21.0)	0.09	18.2 (12.6, 24.3)	13.4 (8.7, 20.6)	0.034
Glucose (mmol/L)	5.0 (4.6, 5.4)	4.9 (4.6, 5.28)	0.65	4.95 (4.7, 5.38)	4.9 (4.57, 5.3)	0.48
Insulin (pmol/L)	37.8 (27.4, 60.5)	35.0 (26.4, 46.9)	0.29	49.2 (28.4, 73.3)	35.0 (26.9, 44.5)	0.0084
HOMA	1.20 (0.78, 1.83)	1.03 (0.79, 1.48)	0.31	1.36 (0.86, 2.51)	1.00 (0.77, 1.42)	0.027
hsCRP (mg/L)	1.6 (0.6, 3.6)	1.0 (0.5, 2.1)	0.045	1.4 (0.7, 3.6)	1.2 (0.6, 2.1)	0.3
Adiponectin (μg/ml)	4.7 (4.4, 5.4)	5.0 (4.2, 5.6)	0.60	5.0 (4.6, 5.6)	4.8 (4.2, 5.5)	0.12
LDL cholesterol (mmol/L)	3.1 (2.5, 3.8)	2.8 (2.3, 3.6)	0.19	3.1 (2.6, 4)	3.0 (2.3, 3.6)	0.16
HDL cholesterol (mmol/L)	1.4 (1.2, 1.6)	1.4 (1.2, 1.6)	0.68	1.4 (1.2, 1.5)	1.4 (1.2, 1.6)	0.57
Triglycerides (mmol/L)	1.19 (0.91, 1.73)	1.10 (0.88, 1.49)	0.47	1.17 (0.98, 1.94)	1.10(0.87, 1.46)	0.12

^a^*P*-value from testing the null hypothesis of no association, using Wilcoxon rank-sum tests.

### CD68 + CLS-B but not hCLS-B have strong prognostic significance

**Fig. 3:**
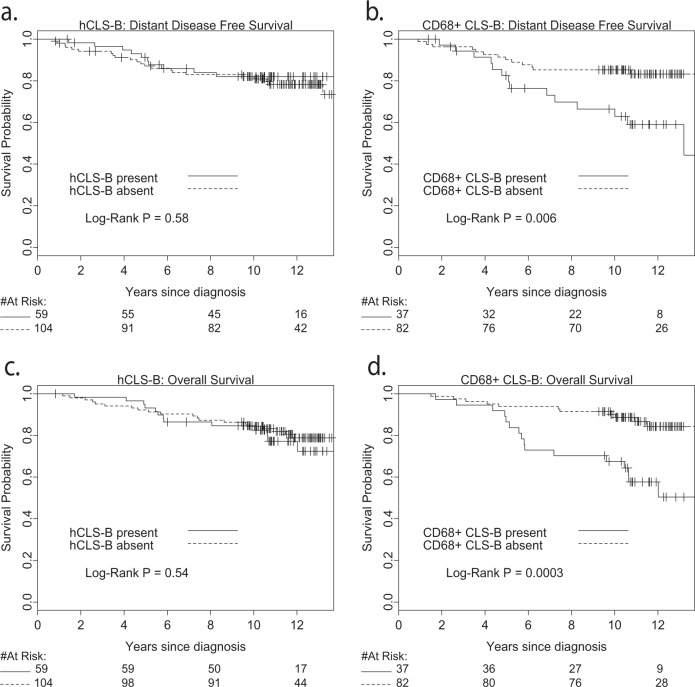
Survival analysis shows prognostic significance of crown-like structures of the breast. Kaplan–Meier distant disease free (**a**, **b**) and overall survival plots (**c**, **d**) for the 163 patients assessed for H&E CLS-B (**a**, **c**) and the subset of 119 assessed by CD68 testing (**b**, **d**).

Kaplan-Meier analysis (Fig. 3) and univariable Cox regression models (Table 4) showed that the presence (vs absence) of CD68 + CLS-B was strongly associated with adverse distant disease-free (HR 2.79; 95% confidence interval (CI) 1.31–5.95, *p* = 0.008) and overall survival (HR 3.74; 95% CI 1.73-8.07, *p* = 0.0008). By contrast, the presence of hCLS-B was not significantly associated with either (DFS HR 0.81; 95% CI 0.38–1.71, *p* = 0.58 and OS HR 1.25; 95% CI 0.62–2.51, *p* = 0.54). Multivariable analysis using Cox proportional hazard models (Table 4) indicated that the associations of CD68 + CLS-B with DFS and OS were not altered after adjustment for clinicopathologic factors, including tumour and nodal stage, grade, ER/PgR status and treatment (surgery, chemotherapy, hormone therapy). The associations of CD68 + CLS-B with OS were also not altered by further adjustment for BMI and metabolic factors (including insulin and hsCRP) (fully adjusted HR 3.58; 95% CI 1.32–9.77, *p* = 0.013). Associations of CD68 + CLS-B with DFS were also not impacted by adjustment for clinicopathologic factors (HR 2.81; 95% CI 1.20–6.57, *p* = 0.017). In contrast, associations of CD68 + CLS-B with DFS were reduced and non-significant after adjustment for BMI and metabolic factors (fully adjusted HR 2.04 95% CI 0.78–5.35, *p* = 0.14). Adjustment for BMI alone had little impact on any of these associations (Table 4). The presence of hCLS-B was not significantly associated with distant recurrence or death after adjustment for clinicopathologic factors (DFS HR 0.59; 95% CI 0.26–1.30, *p* = 0.19 and OS HR 1.04; 95% CI 0.50–2.16, *p* = 0.92) or clinicopathologic factors, BMI and metabolic factors (DFS HR 0.46; 95% CI 0.18–1.19, *p* = 0.11 and OS HR 0.71; 95% CI 0.31–1.65, *p* = 0.43).

**Table 4:** Multivariable Cox proportional hazard models for presence vs. absence of hCLS-B and CD68+CLS-B.

	Presence vs. absence of hCLS-B^a^	Presence vs. absence of hCLS-B^a^	Presence vs. absence of CD68 + CLS-B^a^	Presence vs. absence of CD68 + CLS-B^a^
Model	Distant recurrence	Death	Distant recurrence	Death
Unadjusted	0.81 (0.38, 1.71)*P* = 0.58	1.25 (0.62, 2.51)*P* = 0.54	2.79 (1.31, 5.95)*P* = 0.008	3.74 (1.73, 8.07)*P* = 0.0008
Adjusted for BMI	0.56 (0.25, 1.25)*P* = 0.16	0.76 (0.35, 1.61)*P* = 0.47	2.44 (1.13, 5.27)*P* = 0.022	2.89 (1.32, 6.35)*P* = 0.0083
Adjusted for clinicopathologic characteristics^b^	0.59 (0.26, 1.30)*P* = 0.19	1.04 (0.50, 2.16)*P* = 0.92	2.81 (1.20, 6.57)*P* = 0.017	3.97 (1.66, 9.48)*P* = 0.0019
Adjusted for BMI, insulin, hsCRP and clinicopathologic characteristics^b^	0.46 (0.18, 1.19)*P* = 0.11	0.71 (0.31, 1.65)*P* = 0.43	2.04 (0.78, 5.35)*P* = 0.14	3.58 (1.32, 9.77)*P* = 0.013

^a^Table entries are presented as hazard ratio (95% confidence interval), followed by *P* value. The *P*-value from testing the null hypothesis that the hazard ratio for presence versus absence of CLS-B in Cox model is one.

^b^Age, T- and N-stage, tumour grade, ER/PgR status, adjuvant hormones and chemotherapy, surgical treatment.

### Quantification of CD68 + CLS-B does not contribute to prognostic significance

Among the 37/119 cases in the CD68 + CLS-B group, the majority (21/37, 57%) had only 1 CD68 + CLS-B found within the non-neoplastic adipose tissue. CD68 + CLS-B numbered 2 or 3 in 9/37 (24%) cases, and >4 (range 4 to 15) in 7/37 (19%) cases. There were 8, 5 and 1 distant recurrences and 9, 6 and 1 deaths in these 21, 9 and 7 patients. Using log-rank tests, the *P*-values for no difference among the three groups were 0.24 and 0.18 for distant recurrence and death respectively. Furthermore, the group with 2–3 CD68 + CLS-B had more and the group with 4–15 CD68 + CLS-B had fewer than expected events, so there is little evidence of a missed monotone relationship.

## Discussion

We have shown that CLS-B, whether found on H&E histologic sections (hCLS-B) or as previously reported, using the pan-macrophage marker CD68, occur frequently in breast adipose tissue of breast cancer patients (seen in 36% and 31% of patients, respectively). In a cohort described by Morris et al.^5^, 14 of 30 patients (47%) had CD68 + CLS-B. In contrast to our CD68-stained group, the Morris cohort had a higher average BMI (27.8 vs. 24.7).

In our cohort, both hCLS-B and CD68 + CLS-B were associated with higher weight, BMI and waist circumference. However, associations with metabolic markers and breast cancer outcomes differed. CD68 + CLS-B were associated with higher insulin, HOMA and leptin levels and with increased risk of distant recurrence and death but not with hsCRP, a systemic marker of inflammation. In contrast, hCLS-B were associated with modestly higher hsCRP but not with insulin, HOMA or leptin levels; they were also not associated with an increased risk of distant recurrence or death. These observations suggest that the presence of CD68 + CLS-B vs hCLS-B may reflect different biologic responses to obesity – the former characterised by insulin resistance and poor breast cancer outcomes and the latter by metabolic health with no evidence of adverse outcomes. Further research to determine whether similar patterns associations of CD68 + CLS-B and hCLS-B with metabolic status occur in other populations is needed. Additional research is also needed to understand the nature of the cells present in hCLS-B.

To date, other prospective studies have had conflicting results. Koru-Sengul et al report a cohort (*N* = 150)^17^ showing adverse prognosis associated with CLS-B (HR for overall survival of 12.15 on univariate analysis), whereas Maliniak et al. reported a cohort (*N* = 342)^16^ with no evidence of prognostic significance for CLS-B (HR of 1.02 for overall survival and 0.99 for progression-free survival). One key difference in these reports is the use of CD40 immunohistochemistry for CLS-B analysis by Koru-Sengul et al.^17^, which is an investigational marker for M1 (pro-inflammatory) macrophages. In our series, CD68 + CLS-B were associated with poor DFS (HR = 2.79) and OS (HR = 3.74) independent of BMI; the association with DFS was present after adjustment for BMI but was weaker and no longer statistically significant after further adjustment for insulin and clinicopathologic characteristics, suggesting that these blood markers may be in the biologic pathway mediating CD68 + CLS-B effects on recurrence. In contrast, we observed that the adjusted hazard ratio estimates for hCLS-B for both distant recurrence and death were consistently <1.0 after adjustment for metabolic factors although the association was not statistically significant. Thus, it appears that hCLS-B and CD68 + CLS-B may represent different cellular responses to increased adiposity and, as noted above, have different health implications. Our results therefore are more consistent with earlier retrospective analyses^5,15,23^ suggesting a prognostic significance when CLS-B are present.

CD68 is a marker related to lysosomal glycoproteins, and is generally expressed in human monocytes and macrophages^24,25^. Macrophages express CD68 strongly; however, CD68 may be weaker in immature macrophages which demonstrate low-level lysozyme secretion^26^. There is growing appreciation that macrophages have a spectrum of diverse characteristics and lie on a range from pro-inflammatory (classically activated) M1 macrophages to anti-inflammatory (alternatively activated) M2 macrophages^27,28^. In general, pro-inflammatory M1 macrophages have been associated with adipose-tissue inflammation, insulin resistance and diabetes while anti-inflammatory M2 macrophages have been associated with insulin sensitivity and metabolic health^27^. In cancer microenvironments, pro-inflammatory M1 TAMS have been associated with improved outcomes while anti-inflammatory M2 TAMS have been associated with poor outcomes^28^. The observed association with insulin resistance would suggest that the obesity-associated CD68 + CLS-B we identified in our patients contained classically activated, pro-inflammatory M1 macrophages (reflected by the metabolic associations). The observed association with adverse prognosis suggests they may also have also contained alternatively activated anti-inflammatory M2 macrophages or that it is the systemic changes that lead to poor prognosis rather than the local adipose tissue cellular component. It is also possible that anti-inflammatory M2 activated macrophages (that enhanced tumour invasion and metastasis) leading to poor prognosis were present elsewhere in the tumour microenvironment of these obese subjects but not in the CD68 + CLS-B in the adipose tissue distant from the tumour that we examined. The report by Koru-Sengul and colleagues^17^ also raises the possibility that M1 macrophages in CLS-B are paradoxically associated poor cancer outcomes. We were not able to distinguish between these two possibilities as we did not examine TAMS nor did we differentiate M1 vs M2 activated macrophages.

From a mechanistic standpoint, adipokines (e.g. leptin, adiponectin) and cytokines (IL-6, IL-1β, TNF-α) have been implicated in macrophage-mediated inflammation, and these macrophages in turn upregulate aromatase and AKT/mTOR activation^7,8^. Our findings are consistent with the prevailing model, that the presence of CD68 + CLS-B represents a phenotype linking obesity, macrophage-mediated inflammation and breast cancer^6–8,14^. Because it is difficult to measure localised levels of cytokines directly in patient tissues, it remains unclear whether the macrophages forming CLS-B are a by-product of locally increased cytokines/adipokines, or a direct participant in generating tumourigenic factors.

We found that routine H&E evaluation (compared to CD68 immunohistochemistry) results in a slightly higher detection rate of CLS-B (39% vs 31% in the CD68-stained cohort), and that the CLS-B detected by H&E alone lack the strong prognostic significance of CD68 + CLS-B. The lack of association of hCLS-B with metabolic factors (apart from a weak association with hsCRP that was of borderline significance) and the potential for a protective association with DFS and OS (particularly after adjustment for BMI and metabolic factors) were unexpected. It remains unclear what the cells comprising CD68-negative hCLS-B represent, given that they are morphologically indistinguishable from macrophages. We were not able to characterise these macrophage-like cells further. The findings raise the possibility that hCLS-B represent a lysozyme-poor or immature macrophage subpopulation^26^. We cannot exclude that these may be morphologic mimics of “classical” CLS-B, composed for example of stromal cells or lymphoplasmacytoid inflammatory cells. One limitation of our analysis is that we have used representative sections for CD68 staining (see also below), resulting in less overall sensitivity for CD68 immunostaining. Nevertheless, hCLS-B can be identified by pathologist review using readily applied criteria, and therefore represent a detectable structural change in adipose tissue associated with obesity. This issue should be explored in future research as it is possible these hCLS-B reflect a different patient response to obesity that has a favourable effect on breast cancer outcomes and metabolic state.

Prior reports have also suggested that the number or density of CD68 + CLS-B may correlate with patient outcome, and have proposed various systems of quantifying CLS-B in tissue sections^5,15,23^. In our multivariate analysis, the presence of CD68 + CLS-B (without quantitation) was associated with worse OS (but not DFS) when adjusted for patient and tumour characteristics such as age, BMI, tumour grade and tumour stage. The addition of quantitative measures, such as counts of CLS-B and number of slides involved, did not add independent prognostic information. One limitation of our approach is that we did not attempt to use morphometry to quantify CLS-B density in terms of CLS-B per unit-area of adipose tissue. Our focus has been on data readily obtainable by routine pathologic review. Taken together, our study and prior reports^5,15,23^ therefore do not clearly demonstrate a prognostic significance for CLS-B quantitation. Further prospective investigation appears warranted to evaluate for this possibility.

The optimal method for investigating CLS-B in pathology specimens remains unclear. Dannenberg and colleagues have previously studied CD68 + CLS-B in breast cancer patients, reporting a pilot study (*N* = 30) which showed a CD68 + CLS-B prevalence of 50%^5^, and a subsequent cross-sectional sample (*N* = 100) which showed a CD68 + CLS-B prevalence of 52%^15^. In the latter study (*N* = 100), evaluation of CLS-B was performed on a total of 5 CD68-stained adipose-tissue sections for each case^15^ and correlation to patient outcome was not reported. We examined one representative CD68-stained section per case, showing clear adverse prognosis when CLS-B were detected. This comparison suggests that staining four additional blocks does increase the sensitivity of CLS-B detection (by approximately 50%), but without evidence of gain in prognostic information.

The study of CLS-B requires the examination of adipose tissue away from skin, tumours and other confounding inflammation such as fat necrosis. This is in contrast to related but distinct phenomena such as tumour-associated macrophages^13^ and subcutaneous macrophage inflammation^9^. In our cohort, the number of blocks stained in each case was opportunistic and depended on the number of archival white adipose tissue blocks submitted at the time of routine gross pathologic evaluation. In our series, only 1 block was selected for CD68 immunohistochemistry based on pathologist judgement that attempted to maximise the amount of white-adipose-tissue per block immunostained. Nevertheless, CLS-B detected by this method had strong prognostic significance, implying that no special pathologic processing other than judicious CD68 immunostaining need to be performed to identify clinically significant CLS-B.

One limitation of our method, and of prior reports, is that the study design is purely observational. As a result, there remains no clinical utility for CLS-B with respect to treatment selection or patient monitoring. Our study supports the model that CLS-B represents a host-tissue risk factor localised to the adipose-tissue microenvironment, rather than a tumour factor or direct driver of poor breast cancer outcome. There is emerging evidence that the prevalence of CLS-B may vary with racial differences^17,29^; our data do not address racial disparities. Further work is warranted to determine whether some of these inflammatory mechanisms can be modified to improve cancer outcomes.

In this prospectively followed cohort of breast cancer patients, the presence of crown-like structures of the breast (CLS-B, rings of macrophages in adipose tissue detected by the pan-macrophage marker CD68 immunohistochemistry or by routine H&E staining) was associated with obesity; the presence of CD68 + CLS-B was also associated with insulin resistance and adverse prognosis while the presence of hCLS-B was associated with metabolic health without adverse prognosis. This adverse prognostic association of CD68 + CLS-B was independent of BMI and tumour factors such as grade, stage and hormone receptor status, but was attenuated when adjusted for metabolic factors. These differing metabolic responses, and associated prognostic associations, for CD68 + CLS-B and hCLS-B may reflect differences in macrophage maturity and diversity that are independent of BMI, or potentially other factors not investigated here, including the occurrence of cells other than macrophages in the hCLS-B. Further research is needed to replicate these results and to understand the biologic basis for the differing tissue and metabolic responses to obesity.

## Methods

### Cohort

Patients without known diabetes, and with surgically resected early breast cancer (T1 to T3, N0 or N1, M0), were enroled into a prospective observational study of obesity related factors, including fasting insulin and adipokines, treatment and outcome between 1988 and 1995 (*N* = 535)^20^. Informed consent was obtained from all participants for both the original prospective trial, and for the use of all data in future research-ethics-board-approved research. No public registration was required for this study. These patients were characterised in detail with respect to body mass index (BMI) and fasting metabolic factors^20,21^. Subjects were prospectively followed for a median of 12.1 years (range 0.23–17 years). We had access to archival H&E slides from one of the sites of enrolment of this cohort (Study Site 2 - Mount Sinai Hospital, Toronto, *N* = 163, Fig. 1), which we retrieved for pathologic evaluation in accordance with a protocol approved by the Mount Sinai Hospital institutional Research Ethics Board (Toronto, Canada).

### Review of H&E sections for histologic CLS-B

Archival hematoxylin-and-eosin (H&E) stained slides from routine pathology evaluation were retrieved. All available sections of white adipose tissue without tumour on the same tissue block (median 7 blocks/case), excluding fat necrosis and mastitis, were reviewed by at least 1 of 2 board-certified anatomic pathologists with subspecialty experience in breast cancer (ZE or MC), blinded to blood markers, BMI and survival outcomes. Cases were reviewed in two groups. The first group (*N* = 99) was scored by both study pathologists independently, and discrepancies were resolved by a subsequent consensus review. During consensus review, a final decision was made on each discrepant case, and consensus morphologic criteria for CLS-B were agreed upon. These criteria were: presence of a ring of mononuclear cells surrounding an adipocyte vacuole, mononuclear cells of CLS-B having reniform nuclei consistent with macrophages, absence of tumour, absence of fat necrosis and absence of vascular lumens. In the remainder of the cases (*N* = 64), these criteria were applied by a reviewing pathologist independently.

Although CLS-B as originally described^5,6,15^ are found using CD68 immunohistochemistry (see below), their morphologic counterparts could also be visualised by H&E evaluation (Fig. 2a, c). Structures meeting the morphologic criteria for CLS-B (above) were thus identified, and we refer to these distinctly from CD68-detected (“classical”) CLS-B as “hCLS-B”. The presence and numbers of hCLS-B were recorded.

### CD68 immunohistochemistry

Out of 163 potential cases, archival paraffin blocks were available in a subset of 119 patients (Fig. 1). For each case where at least one hCLS-B was seen, the block corresponding to an H&E section with the highest number of hCLS-B was selected for further staining. For cases in which no hCLS-B were detected, a paraffin block corresponding to the slide with the most abundant white adipose tissue sample was selected for further staining. For each of these selected blocks, a single 4-μm-thick section was cut and placed on positive-charge-coated slides and stained for CD68 (PG-M1 clone, Dako Canada, Burlington, ON, Canada, at 1:75 dilution). The antibody was applied after antigen retrieval at 100 °C for 15 min, and visualised using horseradish-peroxidase-labelled protein polymer and 3,3′-diaminobenzidine as the chromogen (MACH4 system, Biocare Medical, Concord, CA). We refer to CLS-B identified via a positive CD68 stain (e.g., Fig. 2b) as either “CD68 + CLS-B” or simply “CLS-B” to distinguish them from hCLS-B which are found by H&E examination alone.

### Blood assays

Blood was drawn into tubes containing EDTA anticoagulant after an overnight fast of at least 12 h and centrifuged at 1500 rpm for 10 min; plasma was aliquoted and frozen within 30 min of collection at −70 °C. Assays were performed blinded to treatment allocation with 10% random repeats for insulin (Beckman Coulter Access Immunoassay system using the manufacturer’s 2-epitope immunimetric chemiluminescent method), leptin (Linco human leptin kit catalogue No EZ HL-81k solid phase, enzyme linked immunosorbent assay with a limit of sensitivity of 0.5 ng/ml) and hsCRP (Roche, particle based immunoturbidimetric assay). Intra-assay coefficients of variability were 3%, 5% and 4% for insulin, leptin and hsCRP respectively. Glucose was analysed using the enzymatic reference method with hexokinase. Homeostasis Model Assessment (HOMA), a marker of insulin resistance^22^) was calculated from glucose and insulin [glucose (mg/dl) × insulin (pmol/L)/22.5].

### Statistical analysis

The occurrence of hCLS-B versus CD68 + CLS-B was cross-tabulated. Patient and tumour characteristics were summarised, broken down by the presence (versus absence) of each of the two patterns of CLS-B. The hypothesis of no association between a characteristic and the presence of CLS-B was tested using *χ*^2^ tests. Measurements of body size and metabolic factors were summarised for CLS-B and hCLS-B using medians, 25th and 75th percentiles and the hypothesis of no association tested using Wilcoxon rank-sum tests. Kaplan–Meier plots with log-rank tests were used to summarise distant disease free and overall survival related to the presence versus absence of CLS-B or hCLS-B. Cox proportional hazard models were used to examine the effects of adjusting for combinations of BMI, insulin, hsCRP and the patient and tumour characteristics age, T- and N-stage, tumour grade, ER/PgR status, adjuvant hormones and chemotherapy, and surgical treatment.

### Reporting summary

Further information on research design is available in the Nature Research Reporting Summary linked to this article.

## Supplementary information

Supplementary Information

Reporting Summary

## Data Availability

The data generated and analysed during this study are described in the following data record: 10.6084/m9.figshare.14779527^30^. All data underlying the claims of the article are contained in the tab-delimited text file ‘NRF database.txt’. These data are housed on institutional storage and are not publicly available in order to protect patient privacy, as no Ethics approval to share data was received. However, the data will be made available upon request to individual investigators. Data enquiries should be addressed to the corresponding author.
