# Supplementary material for: Crown-like structures in breast adipose tissue of breast cancer patients: associations with CD68 expression, obesity, metabolic factors and prognosis
Source: NPJ Breast Cancer. 2021 Jul 22;7:97. doi: 10.1038/s41523-021-00304-x (PMC8298396; doi:10.1038/s41523-021-00304-x)
Supplement: Supplementary file 1 — Supplementary Information [file 41523_2021_304_MOESM1_ESM.pdf]

**Supplementary Table 1. Comparison of patient and tumour characteristics according to paraffin block availability.**

| | Paraffin blocks Available n=119 | Paraffin blocks Not Available[1] n=44 | P[2] |
|---|---|---|---|
| **Age at diagnosis** | | | 0.4 |
| **≤ 60** | 84 (70.6%) | 34 (77.3%) | |
| **> 60** | 35 (29.4%) | 10 (22.7%) | |
| **Menopausal Status** | | | 0.06 |
| **Pre- or Peri-** | 56 (47.1%) | 28 (63.6%) | |
| **Post** | 63 (52.9%) | 16 (36.4%) | |
| **BMI (kg/m$^2$)** | | | 0.37 |
| **≤ 25** | 61 (51.3%) | 28 (63.6%) | |
| **> 25-30** | 37 (31.1%) | 10 (22.7%) | |
| **> 30** | 21 (17.6%) | 6 (13.6%) | |
| **Surgical Treatment** | | | 0.37 |
| **Total Mastectomy** | 27 (22.7%) | 13 (29.5%) | |
| **Partial Mastectomy** | 92 (77.3%) | 31 (70.5%) | |
| **Tumor Grade (Nottingham)** | | | 0.75 |
| **Grade 1** | 31 (26.1%) | 12 (27.3%) | |
| **Grade 2** | 44 (37%) | 19 (43.2%) | |
| **Grade 3** | 40 (33.6%) | 11 (25%) | |
| **Unknown** | 4 (3.4%) | 2 (4.5%) | |
| **Tumor Stage** | | | 0.96 |
| **pT1** | 69 (58%) | 25 (56.8%) | |
| **pT2** | 36 (30.3%) | 13 (29.5%) | |
| **pT3** | 8 (6.7%) | 4 (9.1%) | |
| **Unknown** | 6 (5%) | 2 (4.5%) | |
| **Nodal Stage** | | | 0.99 |
| **pN0** | 81 (68.1%) | 30 (68.2%) | |
| **pN1-3** | 38 (31.9%) | 14 (31.8%) | |
| **Hormone Receptor** | | | 0.27 |
| **Positive** | 88 (73.9%) | 33 (75%) | |
| **Negative** | 11 (9.2%) | 7 (15.9%) | |
| **Unknown** | 20 (16.8%) | 4 (9.1%) | |
| **Lymphovascular Invasion** | | | 0.85 |
| **Present** | 22 (18.5%) | 9 (20.5%) | |
| **Absent** | 60 (50.4%) | 20 (45.5%) | |
| **Unknown** | 37 (31.1%) | 15 (34.1%) | |

1. Paraffin blocks were needed in order to do CD68 staining.
2. P-value from testing the null hypothesis of no association, using $\chi 2$ tests.
