# Supplementary material for: Crown-like structures in breast adipose tissue of breast cancer patients: associations with CD68 expression, obesity, metabolic factors and prognosis
Source: NPJ Breast Cancer. 2021 Jul 22;7:97. doi: 10.1038/s41523-021-00304-x (PMC8298396; doi:10.1038/s41523-021-00304-x)
Supplement: Supplementary file 2 — Reporting Summary [file 41523_2021_304_MOESM2_ESM.pdf]

nature research

Corresponding author(s): CHANG, Martin C.

Last updated by author(s): May 4, 2021

Reporting Summary

Nature Research wishes to improve the reproducibility of the work that we publish. This form provides structure for consistency and transparency in reporting. For further information on Nature Research policies, see our Editorial Policies and the Editorial Policy Checklist.

Statistics

For all statistical analyses, confirm that the following items are present in the figure legend, table legend, main text, or Methods section.

n/a	Confirmed	
☐	☒	The exact sample size (n) for each experimental group/condition, given as a discrete number and unit of measurement
☒	☐	A statement on whether measurements were taken from distinct samples or whether the same sample was measured repeatedly
☐	☒	The statistical test(s) used AND whether they are one- or two-sided<br>*Only common tests should be described solely by name; describe more complex techniques in the Methods section.*
☐	☒	A description of all covariates tested
☐	☒	A description of any assumptions or corrections, such as tests of normality and adjustment for multiple comparisons
☐	☒	A full description of the statistical parameters including central tendency (e.g. means) or other basic estimates (e.g. regression coefficient) AND variation (e.g. standard deviation) or associated estimates of uncertainty (e.g. confidence intervals)
☐	☒	For null hypothesis testing, the test statistic (e.g. F, t, r) with confidence intervals, effect sizes, degrees of freedom and P value noted<br>*Give P values as exact values whenever suitable.*
☒	☐	For Bayesian analysis, information on the choice of priors and Markov chain Monte Carlo settings
☒	☐	For hierarchical and complex designs, identification of the appropriate level for tests and full reporting of outcomes
☒	☐	Estimates of effect sizes (e.g. Cohen's d, Pearson's r), indicating how they were calculated

Our web collection on statistics for biologists contains articles on many of the points above.

Software and code

Policy information about availability of computer code

Data collection	Baseline data collection started in 1989 using a SIR (public access) database; data were transferred to SAS (SAS Institute) 6.12 in 1999. Follow-up data was collected in a Microsoft Access 97 database. Data were transferred to updated versions of the software as these became available. There was no use of non-standard features or custom code.
Data analysis	Data analysed with the assistance of Microsoft Excel 2010-2016, SAS (SAS Institute) 9.2 and S-PLUS (Tibco) 6.2. There was no use of non-standard features or custom code.

For manuscripts utilizing custom algorithms or software that are central to the research but not yet described in published literature, software must be made available to editors and reviewers. We strongly encourage code deposition in a community repository (e.g. GitHub). See the Nature Research guidelines for submitting code & software for further information.

Data

Policy information about availability of data

All manuscripts must include a data availability statement. This statement should provide the following information, where applicable:

- Accession codes, unique identifiers, or web links for publicly available datasets
- A list of figures that have associated raw data
- A description of any restrictions on data availability

The datasets generated during and/or analysed during the current study are available from the corresponding author on reasonable request.

Field-specific reporting

Please select the one below that is the best fit for your research. If you are not sure, read the appropriate sections before making your selection.

☒ Life sciences ☐ Behavioural & social sciences ☐ Ecological, evolutionary & environmental sciences

For a reference copy of the document with all sections, see nature.com/documents/nr-reporting-summary-flat.pdf

Life sciences study design

All studies must disclose on these points even when the disclosure is negative.

Sample size	Determined from availability of tissue samples from a legacy cohort. No additional prospective sample size determinations were needed.
Data exclusions	No data were excluded from the analysis
Replication	Interobserver replicability was addressed by consensus review by the study pathologists. Replication of CD68 antibody staining was assured by control staining on an automated platform.
Randomization	The clinical data were obtained using a prospective cohort method, without a need for random allocation.
Blinding	Investigators were blinded to cohort characteristics when analysing the data under investigation in this manuscript.

Reporting for specific materials, systems and methods

We require information from authors about some types of materials, experimental systems and methods used in many studies. Here, indicate whether each material, system or method listed is relevant to your study. If you are not sure if a list item applies to your research, read the appropriate section before selecting a response.

Materials & experimental systems

n/a	Involved in the study	
☐	☒	Antibodies
☒	☐	Eukaryotic cell lines
☒	☐	Palaeontology and archaeology
☒	☐	Animals and other organisms
☐	☒	Human research participants
☐	☒	Clinical data
☒	☐	Dual use research of concern

Methods

n/a	Involved in the study	
☒	☐	ChIP-seq
☒	☐	Flow cytometry
☒	☐	MRI-based neuroimaging

Antibodies

Antibodies used	CD68 (immunohistochemistry, clone PG-M1, Dako Canada, Catalog No. M0876, Multiple lots).
Validation	This is a Health Canada approved in vitro diagnostic, validated on control human tissues containing macrophages (at least 20 cases with any change in protocol) in a CLIA certified clinical laboratory.

Human research participants

Policy information about studies involving human research participants

Population characteristics	The population comprised a consecutive cohort of women who underwent treatment for operable breast cancer at Mount Sinai Hospital (Toronto, Canada) between June 1989 and June 1996. Women were included if they had age less than 75 years, complete resection of breast cancer and axillary dissection for previously untreated breast cancer. Women were excluded if they had prior malignancy (except cervical in situ lesion or nonmelanoma skin cancer), a serious coexisting medical condition, including diabetes, use of medications that modified key study variables, or inability to provide consent.
Recruitment	Consecutive women were recruited from those women undergoing surgery for breast cancer (see above). Women were excluded if they had prior malignancy (except cervical in situ lesion or nonmelanoma skin cancer), a serious coexisting medical condition, including diabetes, use of medications that modified key study variables, or inability to provide consent. No systematic source of bias was identified.
Ethics oversight	Mount Sinai Hospital, Toronto, Canada, Research Ethics Board

Note that full information on the approval of the study protocol must also be provided in the manuscript.

Clinical data

Policy information about clinical studies

All manuscripts should comply with the ICMJE guidelines for publication of clinical research and a completed CONSORT checklist must be included with all submissions.

Clinical trial registration	This was a prospective observational trial. The enrollment completed in 1996. Therefore, no clinical trials registration was required or obtained.
Study protocol	The original study protocol was published in 2002. (PMID: 11773152)
Data collection	The population comprised a consecutive cohort of women who underwent treatment for operable breast cancer at Mount Sinai Hospital (Toronto, Canada) between June 1989 and June 1996.
Outcomes	Overall survival and Disease-free survival (breast cancer) were the primary outcomes, obtained by patient follow-up.
